# Effects of low-level laser therapy on the orthodontic mini-implants stability: a systematic review and meta-analysis

**DOI:** 10.1186/s40510-021-00350-y

**Published:** 2021-02-15

**Authors:** Ana Carolina de Figueiredo Costa, Thays Allane Cordeiro Maia, Paulo Goberlânio de Barros Silva, Lucas Guimarães Abreu, Delane Viana Gondim, Pedro César Fernandes Santos

**Affiliations:** 1 Department of Clinical Dentistry, Faculty of Pharmacy, Dentistry and Nursing, Federal University of Ceará, Monsenhor Furtado Street, Rodolfo Teófilo, Fortaleza, Ceará 60430-350 Brazil; 2 Department of Paediatric Dentistry and Orthodontics, Faculty of Dentistry, Federal University of Minas Gerais, Belo Horizonte, Minas Gerais Brazil; 3 Department of Morphology, Faculty of Medicine, Federal University of Ceará, Fortaleza, Ceará Brazil

**Keywords:** Orthodontic anchorage procedures, Mini-implants, Low-level laser therapy, Stability

## Abstract

**Objectives:**

The aim of this systematic review and meta-analysis was to assess the effects of low-level laser therapy (LLLT) on the orthodontic mini-implants (OMI) stability.

**Materials and methods:**

An unrestricted electronic database search in PubMed, Science Direct, Embase, Scopus, Web of Science, Cochrane Library, LILACS, Google Scholar, and ClinicalTrials.gov and a hand search were performed up to December 2020. Randomized clinical trials (RCTs) or non-randomized clinical trials (Non-RCTs) that assessed the effects of LLLT on the OMI stability were included. Data regarding the general information, LLLT characteristics, and outcomes were extracted. The authors performed risk of bias assessment with Cochrane Collaboration’s or ROBINS-I tool. Meta-analysis was also conducted.

**Results:**

Five RCTs and one Non-RCT were included and 108 patients were evaluated. The LLLT characteristics presented different wavelength, power, energy density, irradiation time, and protocol duration. Five RCTs had a low risk of selection bias. Two RCTs had a low risk of performance and detection bias. All RCTs had a low risk of attrition bias, reporting bias and other bias. The Non-RCT presented a low risk of bias for all criteria, except for the bias in selection of participants. The meta-analysis revealed that LLLT significantly increased the OMI stability (*p* < 0.001, Cohen’s *d* = 0.67) and the highest clinical benefit was showed after 1 (*p* < 0.001, Cohen’s *d* = 0.75), 2 (*p* < 0.001, Cohen’s *d* = 1.21), and 3 (*p* < 0.001, Cohen’s *d* = 1.51) months of OMI placement.

**Conclusions:**

LLLT shows positive effects on the OMI stability.

**Supplementary Information:**

The online version contains supplementary material available at 10.1186/s40510-021-00350-y.

## Introduction

Orthodontic mini-implants (OMI) are the most effective tool for reinforcement of orthodontic anchorage [1]. This temporary anchorage device has become popular among orthodontists, being considered a versatile, well-tolerated, low-invasive, simple to insert ad low-cost method. Moreover, OMI provides great mechanical predictability and stability [2].

The maintenance of mechanical stability and the absence of pain and peri-implant inflammation are directly related to the clinical success of the OMIs [3, 4]. Primary stability is conferred shortly after the procedure for placement of the device and secondary stability is expressed after the healing phase. The retention of OMI in bone depends on numerous factors, such as bone density and thickness of the insertion site, device surface morphology, surgical technique, and physiological repair process [3, 5, 6].

The low-level laser therapy (LLLT) is a non-invasive and painless method, consisting of the use of non-ionizing light sources of the visible or infrared spectrum that acts under mitochondrial photoreceptors, increasing adenosine triphosphate production and cell proliferation [7]. Recent ex vivo and in vivo studies have demonstrated that the LLLT has promising effects in orthodontics, accelerating orthodontic movement, reducing orthodontic pain, and increasing the primary and secondary stability of OMIs [5, 8–11]. This last topic has great clinical interest, but there are still no systematic reviews aiming to elucidate the true effects of this therapy on skeletal anchorage.

Therefore, the present study aimed to perform a systematic review and meta-analysis of clinical trials assessing the effects of LLLT on the OMI stability.

## Material and methods

### Protocol and registration

This systematic review was submitted to the PROSPERO database (CRD42020188469). The reporting of this study is in accordance with the Preferred Reporting Items for Systematic Reviews and Meta-Analyses (PRISMA) statement and followed the guidelines in the Cochrane Handbook for Systematic Reviews of Interventions.

### Eligibility criteria

The selection criteria were structured according to the PICOS (Patients, Intervention, Control, Outcome, Study design) strategy:

- *Patients (P)*. Individuals of both sexes, in permanent dentition, without restriction on ethnic or socioeconomic group, whose orthodontic treatment with fixed appliances required anchorage with OMI
- *Intervention (I)*. Application of LLLT on OMI
- *Control (C)*. Patients who received placebo or no treatment on OMI
- *Outcome (O)*. The primary outcome included the OMI stability. The secondary outcome could include pain, peri-implant inflammation, clinical success/failure range, or displacement of the OMI.
- *Study design (S)*. Randomized clinical trials (RCTs) or non-randomized clinical trials (Non-RCTs)

Animal and laboratory studies, technical and case reports, and opinion and review articles were excluded.

### Information sources

The main search was performed in the following electronic databases: PubMed, Science Direct, Embase, Scopus, Web of Science, Cochrane Library, and LILACS. Searches were conducted from databases’ date of inception until December 2020. A combination of the Boolean operators AND/OR and MeSH/non-MeSH terms was used to identify pertinent studies. The following search strategy was employed: orthodontic anchorage procedures OR mini-implants OR mini-screws OR micro-implants OR skeletal anchorage OR temporary anchorage device OR bone screws AND lasers OR laser therapy OR low-level light therapy. Unpublished literature was searched in Google Scholar and ClinicalTrials.gov. The reference lists of all eligible studies were hand-searched to identify any additional relevant articles that might have been missed during the searches (Supplementary Material 1).

### Search strategy and study selection

Two independent reviewers (ACFC and TACM) performed the study selection that comprised assessment of title, abstract, and full text of the retrieved references. No language or publication date restriction was imposed. After exclusion of duplicate and non-eligible studies, the full text of references considered eligible for inclusion were assessed by the two reviewers independently. Cases of disagreements were resolved by a third reviewer (PCFS). The Cohen *κ* test was used to evaluate the level of agreement between reviewers (ACFC and TACM).

### Data collection process

The two review authors (ACFC and TACM) extracted the relevant data of the included studies independently. A third reviewer (PCFS) resolved any discrepancies and questions.

Information from the included studies was synthesized by tabulating the general characteristics, including author, year of publication, country where the study was carried out, study design, number of participants along with information on their age and sex, number of OMI placed, site of placement and type of load, groups, evaluation methods, and follow-up. The specific information included the LLLT characteristics [laser device, wavelength, irradiation site, irradiation time/frequency, power (W), energy density (J/cm^2^), applications], the main findings and conclusions of the included studies.

### Risk of bias assessment

The risk of bias of included randomized studies was assessment with the Cochrane Collaboration’s Risk of Bias tool [12]. The criteria analyzed were (1) random sequence generation—selection bias, (2) allocation concealment—selection bias, (3) blinding of participants and personnel—performance bias, (4) blinding of outcome assessment—detection bias, (5) incomplete outcome data—attrition bias, (6) selective reporting—reporting bias, and (7) other bias. The risk of bias of included non-randomized studies was assessment with the Risk of Bias in Non-randomized Studies of Interventions (ROBINS-I) tool [13]. The criteria analyzed were (1) bias due to confounding, (2) bias in selection of participants into the study, (3) bias in measurement of interventions, (4) bias due to departures from intended interventions, (5) bias due to missing data, (6) bias in measurement of outcomes, (7) bias in selection of the reported result, and (8) overall.

Two review authors (ACFC and TACM) performed this qualitative synthesis independently and a third author (PCFS) resolved the discrepancies. The studies were classified in low, high, or unclear risk of bias.

### Meta-analysis

To perform the meta-analysis, the means, standard deviation (SD), and sample size of the studies cited were extracted. Because they are different scales, a meta-analysis was performed by calculating the standardized means difference (SMD) and Cohen’s *d* estimate. As they are negative scales, the values obtained from the periotest value (PTV) had their sign inverted to adapt to the same standard of measurement of the positive scales—resonance frequency analysis (RFA) in Hertz or ISQ-value. Baseline measurements were considered, 3 days, 6-9 days, 12–15 days, 1 month (between 3 and 4 weeks), 2 months (between 6 and 8 weeks), and 3 months (between 10 and 12 weeks) after the OMI insertion. Inverse variance method with random effects was used for all meta-analysis. In both cases, a heterogeneity test and calculation of the *I*^2^ coefficient were performed using the RevMan® software (*p* < 0.05).

## Results

### Study selection

**Fig. 1:**
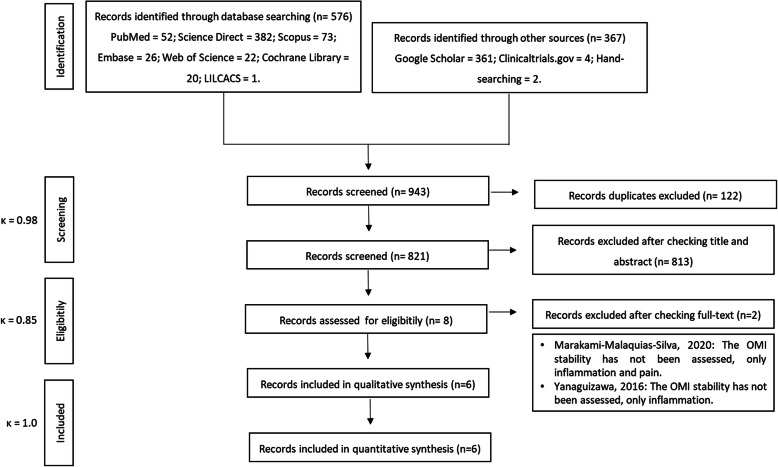
PRISMA flow diagram for systematic reviews and meta-analysis

A total of 943 references was identified in the initial search. After removing duplicates, 821 studies remained. Based on the PICOS strategy defined in this systematic review and met-analysis, 813 studies were excluded after assessment of titles and abstracts. The full texts of eight articles were retrieved and the eligibility was assessed. At the end of the study selection, six studies were included for the qualitative and quantitative synthesis [5, 6, 10, 14–16]. The level of agreement between reviewers was excellent (*ĸ* = 0.98 for screening, *ĸ* = 0.85 for eligibility, and *ĸ* = 1.0 for included) (Fig. 1).

### Risk of bias assessment

**Fig. 2:**
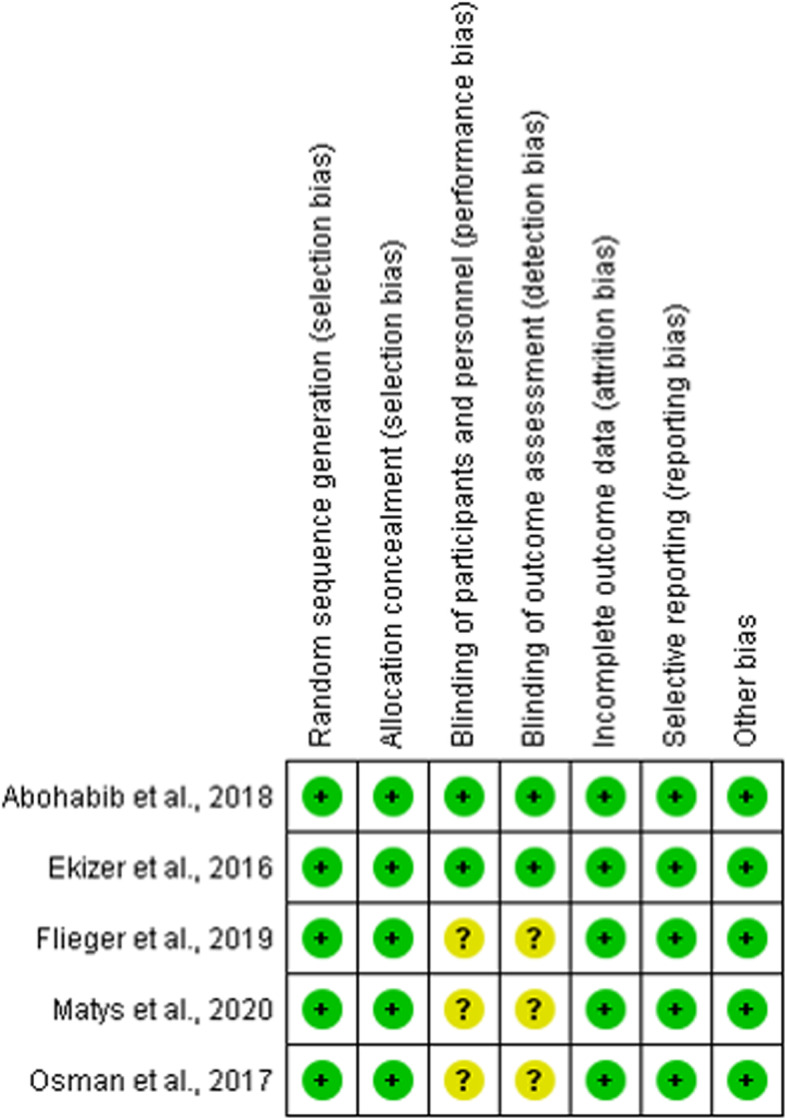
Summary of the risk of bias by Cochrane Collaboration’s tool of included RCTs. The colors indicate the following: low risk of bias (green), high risk of bias (red), or unclear risk of bias (yellow)

**Fig. 3:**
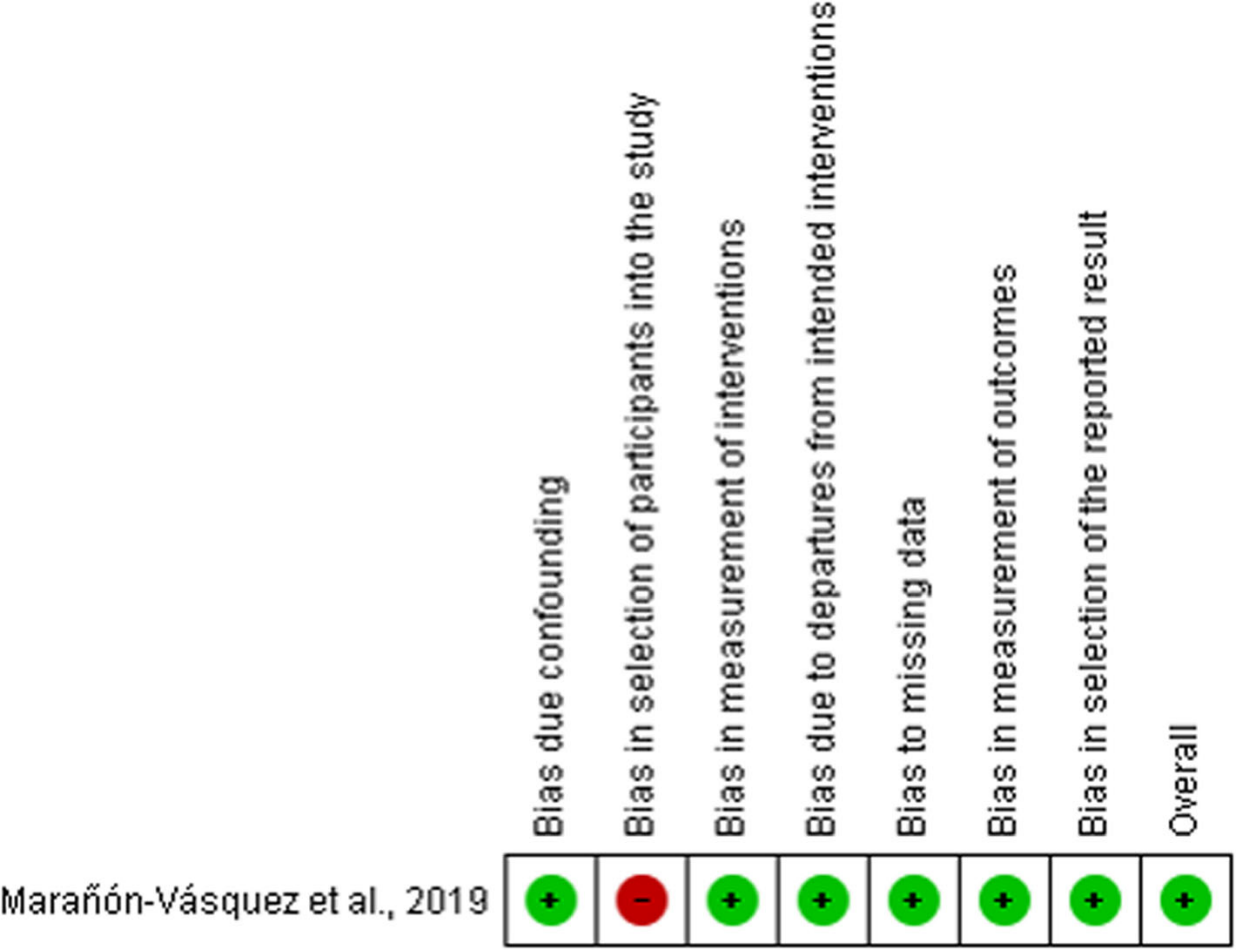
Summary of the risk of bias by ROBBINS-I tool of included Non-RCT. The colors indicate the following: low risk of bias (green) and high risk of bias (red)

By Cochrane Collaboration’s tool, five RCTs had a low risk of selection bias (random sequence generation and allocation concealment) [5, 10, 14–16]. In performance (blinding of participants and personnel) and detection bias (blinding of outcome assessor), two studies had a low risk [10, 14] and three had an unclear risk [5, 15, 16]. All RCTs had a low risk of attrition bias (incomplete outcome data), reporting bias (selective reporting), and other bias [5, 10, 14–16] (Fig. 2). By ROBINS-I tool, the Non-RCT had a low risk of bias for all criteria evaluated, except for bias in selection of participants into the study [6] (Fig. 3).

### Main findings of the systematic review and meta-analysis

All eligible studies were clinical trials published in the past five years [5, 6, 10, 14–16]. Five studies were RCTs [5, 10, 14–16]. Only one study was Non-RCT [6]. The studies were conducted in Egypt [10, 15], Turkey [14], Poland [5, 16], and Brazil [6], involving 108 patients of both sexes between 14 and 32.5 years [5, 6, 10, 14–16]. A total of 206 OMI, with diameter variation of 1.4 and 1.6 mm and length variation of 8 and 10 mm, were exposed to LLLT or placebo [5, 6, 10, 14–16]. Maxilla was the site of placement of the OMIs in six studies [5, 6, 10, 14–16]. The mandible was also a site placement in only one study [6]. The load of 150 g was adopted in three studies [6, 10, 15]. One study evaluated immediate load [10], one study evaluated delay load [15], one study compared both [6], and in three studies, this information was unavailable [5, 14, 16]. All included articles evaluated the OMI stability through the PTV [5, 15, 16] or RFA [6, 10, 14]. The 1/3 evaluated pain [5, 16], 1/3 evaluated local inflammation [14, 15], and 1/3 evaluated parameters related to mobility were also assessed [6, 10]. The follow-up ranged from 1 to 3 months [5, 6, 10, 14–16] (Table 1).

**Table 1:** General characteristics of included studies

Author, year	Country	Study design	Participants (sample; gender; age)	OMI (sample; dimensions; site; load)	Groups	Evaluation methods	Follow-up time
Abohabib, 2018 [10]	Egypt	RCT	15 participants; -; 20.9 ± 3.4 years	30 OMI; 1.5 mm diameter and 8 mm length; maxilla; IL (150 g)	L group; C group	Stability: RFA using the Osstell ISQ device. Clinical success/failure rates: absence/presence of mobility.	10 weeks
Ekizer, 2016 [14]	Turkey	RCT	20 participants; 13 F/7 M; 16.77 ± 1.41 years	20 OMI; 1.6 mm diameter and 8 mm length; maxilla; -	L group; C group	Stability: RFA using the Osstell ISQ device. Inflammation: interleukin-1β levels in gingival and peri-implant crevicular fluid	3 months
Flieger, 2019	Poland	RCT	20 participants; 13 F/7M; 32.5 ± 6.1 years	40 OMI; 1.4 mm diameter and 10 mm length; maxilla; -.	L group; C group	Stability: PTV using the Periotest device. Pain: NRS-11	60 days
Marañón-Vásquez, 2019 [6]	Brazil	Non-RCT	19 participants; -; -	48 OMI for stability; 35 OMI for displacement; 1.5 mm diameter and 8 mm length; maxilla/mandible; IL/DL (150 g)	PBM + IL group; PBM + DL group; IL group/DL group	Stability: RFA using the Osstell ISQ device. Displacement: Images from CBCT	3 months
Matys, 2020 [16]	Poland	RCT	22 participants; 14 F/8 M; 31.7 ± 9.7 years	44 OMI; 1.4 mm diameter and 10 mm length; maxilla; -	L group; C group	Stability: PTV using the Periotest device. Pain: NRS-11	60 days
Osman, 2017 [15]	Egypt	RCT	12 participants; 6 F/6 M; 14 to 28 years	24 OMI; -; maxilla; DL (150 g)	L group; C group	Stability: PTV using the Periotest device. Inflammation: gingival index	60 days

*RCT* randomized clinical trial, *Non-RCT* non-randomized clinical trial, *F* female sex, *M* male sex, OMI orthodontic mini-implant, *L* laser, *C* control, *PBM* photobiomodulation, *IL* immediate loading, *DL* delay loading, *PTV* periotest value, *NRS-11* numeric pain rating scale-11, *RFA* resonance frequency analysis, *CBCT* cone beam computed tomograph

Regarding the LLLT characteristics, the studies used the diode laser with wavelength varying from 618 to 940 nm, power varying from 0.1 to 1.7 W, and energy density varying from 4 to 36 J/cm^2^ [5, 6, 10, 14–16]. The irradiation time per point varied from 20 s to 20 min and the applications duration varied from 14 to 30 days [5, 6, 10, 14–16]. Four studies adopted irradiation at one point under the OMI insertion area [6, 10, 14, 15], and two studies irradiated at two points, by buccal and palatal areas of the maxillary ridge [5, 16] (Table 2).

**Table 2:** LLLT characteristics of the included studies

Author, year	Laser device/wavelength	Irradiation site	Irradiation time/frequency	Power (W)	Energy density (J/cm^**2**^)	Applications
Abohabib, 2018 [10]	Diode laser (Epic 10 Console, Biolase Technology, San Clemente, EUA)/940 nm	Perpendicular to the OMI	60 s/one applications per session	1.7	36	Immediately and 7, 14, 21 days after surgery
Ekizer, 2016 [14]	Diode laser (OsseoPulse 1, Biolux Research Ltd, Vancouver, Canada)/618 nm	OMI insertion area	20 min/one applications per session	0.2	-	Once a day for 21 days
Flieger, 2019	Diode laser (SmartM, Lasotronix, Piazeczno, Poland)/635 nm	Buccal and palatal to the maxillary ridge	100 s per point/two applications per session	0.1	20	Immediately and 3, 6, 9, 12, 15, 30 days after surgery
Marañón-Vásquez, 2019 [6]	Diode laser (Therapy XT, DCM, Kearny, NJ)/660 nm immediately and 808 nm posteriorly	OMI insertion area	20 s (immediately); 40 s (posteriorly)/one applications per session	0.1	4 (immediately); 8 (posteriorly).	Immediately and 2, 4, 7, 9, 11, 14 days after surgery
Matys, 2020 [16]	Diode laser (SmartM PRO, Lasotronix, Warsaw, Poland)/808 nm	Buccal and palatal to the maxillary ridge	40 s per point/two applications per session	0.1	8	Immediately and 3, 6, 9, 12, 15, 30 days after surgery
Osman, 2017 [15]	Diode laser (Epic 10 Console, Biolase Technology, San Clemente, EUA)/910 nm	OMI insertion area	60 s/one applications per session	0.7	-	14 days with an interval of 72 hours between each application (four applications)

*LLLT* low-level laser therapy, *OMI* orthodontic mini-implant, *s* seconds, *min* minutes, *W* watts, *J/cm*^*2*^ Joules per cm^2^

Matys et al. and Flieger et al. observed that LLLT significantly increased the OMI stability, although there was no difference in pain experience [5, 16]. Ekizer et al. also suggested that this therapeutic modality had positive effects on OMI stability, but there was no difference in the interleukin-1β levels in the gingival and peri-implant crevicular fluid [14]. On the other hand, Osman et al. showed that LLLT improved the peri-implant inflammation but, despite improving OMI stability, there was no significant difference [15]. Marañón-Vásquez et al. found that the groups of individuals who received laser irradiation showed a reduction in the loss of the OMI stability and that the delay load increases this effect [6]. Only Abohabib et al. did not recommend the LLLT to promote the OMI stability during canine retraction [10]. This author found that, despite the LLLT reducing the RFA, there was no difference between the success/failure rate between the intervention and control groups [10] (Table 3).

**Table 3:** Outcomes of included studies

Author, year	Main findings	Conclusions
Abohabib, 2018 [10]	The overall success rate of the OMI was 78.5% for L and C groups. The overall failed were three in each group, mainly observed within the first 6 weeks. However, from 3 to 10 weeks, the L group showed significantly increased mean RFA compared to C group.	LLT cannot be recommended as a clinically useful adjunct to promoting OMI stability during canine retraction.
Ekizer, 2016 [14]	OMI stability was significantly increased in the L group after 2 and 3 months. There were no significant differences in the interleukin-1β levels between the groups.	LLLT had a positive effect on OMI stability.
Flieger, 2019	L group showed higher secondary stability (lower mean PTV) in comparison with C group after 3, 30, and 60 days. There was no significant difference in the experience of pain between the groups.	LLLT increased secondary OMI stability.
Marañón-Vásquez, 2019 [6]	PBM groups presented lower loss of stability. DL groups presented lower loss of stability, when the effective period of loading was assessed, independently of the application of PBM. All groups showed displacement of the OMI without significant differences.	DL potentiated the effect of PBM, decreasing the loss of stability.
Matys, 2020 [16]	L group showed higher secondary stability (lower mean PTV) on 30 and 60 days after starting treatment. There was no significant difference in the experience of pain between the groups.	LLLT increased secondary OMI stability.
Osman, 2017 [15]	LLLT improved the stability of OMI, reducing PVT, but the results were not statistically significant. LLLT reduced the gingival index values around OMI, whereas the C group experienced moderate inflammation after 2 months of placement.	LLLT can be suggested as a clinical adjuvant for improving clinical success with OMI.

*LLLT* low-level laser therapy, *OMI* orthodontic mini-implant, *L* laser, *C* control, *PTV* periotest value, *PBM* photobiomodulation, *DL* delay loading, *RFA* resonance frequency analysis

**Fig. 4:**
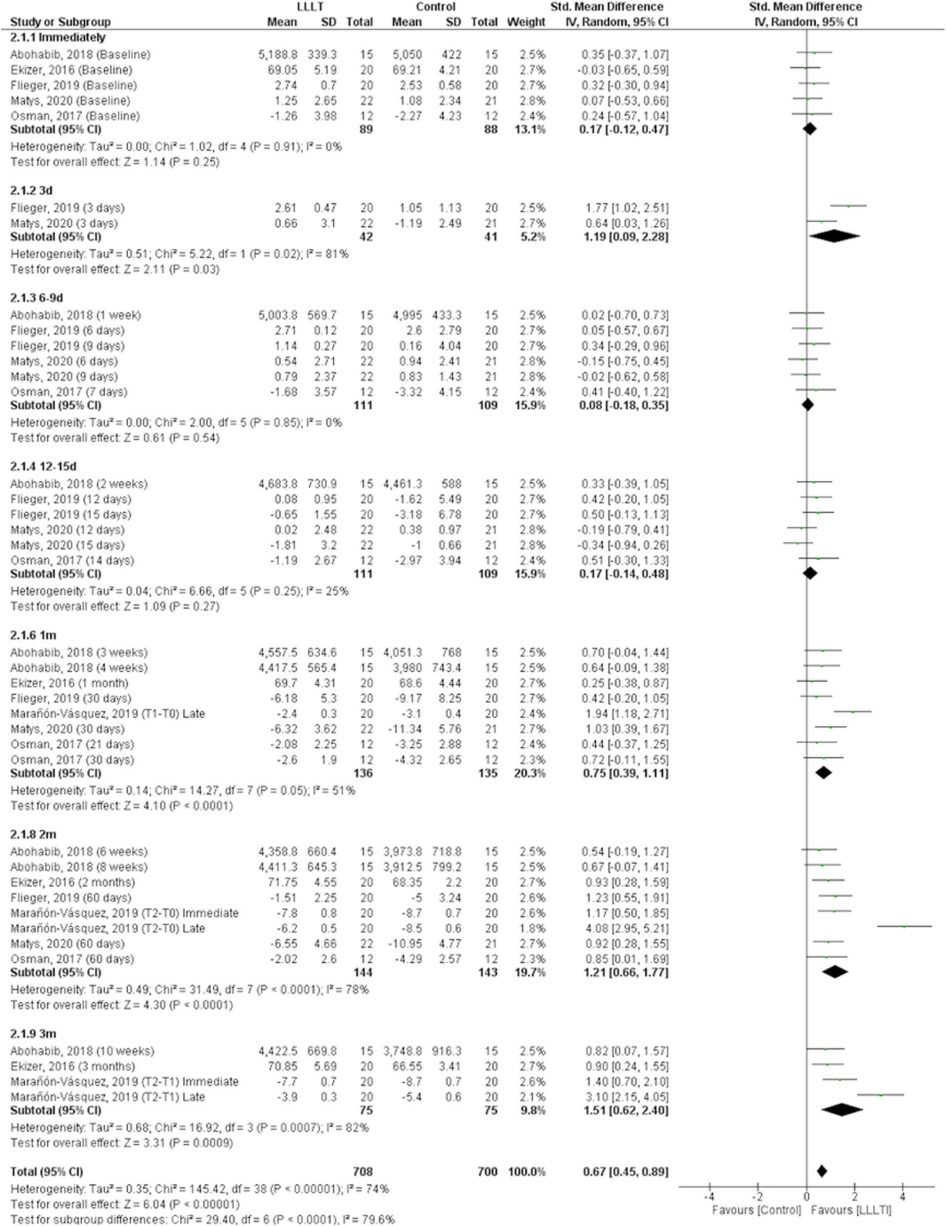
Forest plot of the OMI stability according to the LLLT or control groups. Studies on the left and the right side of the middle line favor the control and the LLLT, respectively

When the OMI stability data was evaluated, it was observed that, even with different scales, the LLLT significantly benefited this outcome (*p* < 0.001) with a Cohen’s *d* value of 0.67 (CI 95% = 0.45 to 0.89), a moderate effect estimate. However, due to the significant heterogeneity between/among studies (*p* < 0.001, *I*^2^ = 74%), subgroups analyses depending on the number of days or months after the LLLT were performed (Fig. 4).

Five studies performed the evaluation immediately after the OMI insertion and applying the LLLT or control [5, 10, 14–16]. There was no significant heterogeneity between/among studies (*p* = 0.910, *I*^2^ = 0%) and there was no significant difference between groups (*p* = 0.250). The sensitivity analysis showed that the removal of studies from the meta-analysis did not significantly modify this outcome (Fig. 4).

The evaluation after 3 days was performed in Flieger et al. and Matys et al. including 42 LLLT patients and 41 control patients [5, 16]. There was significant heterogeneity between the two studies (*p* = 0.020, *I*^2^ = 81%) and treatment with LLLT showed a significant increase in the OMI stability compared to the control group (*p* = 0.030; Cohen’s *d* value = 1.19 (CI 95% = 0.09 to 2.28). The sensitivity analysis showed that the removal of data of Flieger et al. [5] (*p* = 0.040) or Matys et al. [16] (*p* < 0.001) did not change this outcome (Fig. 4).

Between day 6 and day 9 period, four studies evaluating six outcomes showed that there was no significant difference between the LLLT or control in the OMI stability (*p* = 0.540) [5, 10, 15, 16]. There was no significant heterogeneity among the studies (*p* = 0.850, *I*^2^ = 0%). The sensitivity analysis showed that the removal of individual studies did not significantly modify this outcome (Fig. 4).

Between day 12 and day 15, four studies evaluating six outcomes showed that there was no significant difference between the LLLT or control in the OMI stability (*p* = 0.270) [5, 10, 15, 16]. There was no significant heterogeneity among studies (*p* = 0.250, *I*^2^ = 25%) and the sensitivity analysis showed that the individual removal of studies did not significantly modify this outcome (Fig. 4).

One month (between three to four weeks) after OMI placement, all the eligible studies, with a total of eight evaluation moments, showed that treatment with LLLT significantly improved the OMI stability (*p* < 0.001) with a Cohen’s *d* value of 0.75 (CI 95% = 0.39 to 1.11) [5, 6, 10, 14–16]. There was no significant heterogeneity among studies (*p* = 0.050, *I*^2^ = 51%). The sensitivity analysis showed that the removal of individual studies did not significantly modify this outcome (Fig. 4).

Two months of evaluation (between six and eight weeks) after OMI placement, the six studies, with a total of eight evaluation moments, showed that the LLLT significantly improved the OMI stability (*p* < 0.001) with a Cohen’s *d* value of 1.21 (CI05% = 0.66 to 1.77) [5, 6, 10, 14–16]. There was significant heterogeneity among studies (*p* < 0.001, *I*^2^ = 78%). The sensitivity analysis showed that the removal of individual studies did not significantly modify this outcome, except for the removal of data from Marañón-Vásquez et al. [6] (T2-T0), which significantly reduced heterogeneity (*p* = 0.830, *I*^2^ = 0%) (Fig. 4).

Three months (between 10 and 12 weeks) after OMI placement, the three studies with a total of four evaluations showed that treatment with LLLT significantly improved the OMI stability (*p* < 0.001) with a Cohen’s *d* value of 1.51 (CI 95% = 0.62 to 2.40) [6, 10, 14]. There was significant heterogeneity among studies (*p* < 0.001, *I*^2^ = 82%). The sensitivity analysis showed that the removal of individual studies did not significantly modify this outcome, except for the removal of data from Marañón-Vásquez et al. [6] (T2-T1), which significantly reduced heterogeneity (*p* = 0.460, *I*^2^ = 0%) (Fig. 4).

## Discussion

The quantity and quality of systematic reviews in Orthodontics have increased in recent years [17]. However, this is the first systematic review and meta-analysis to elucidate the effects of LLLT on OMI stability. It is also relevant to investigate secondary outcomes such as pain, peri-implant inflammation, clinical success/failure range, or displacement of the OMI, because they are related to stability.

The OMI stability can be assessed clinically by different methods, such as measuring insertion torque, resonance frequency analysis (RFA), and periotest value (PTV) [18]. The last two methods have greater sensitivity to measure the OMI stability and were used by the included articles of this systematic review and meta-analysis [18, 19].

Periotest was originally developed to measure the damping effect of the periodontal ligament around the teeth [20]. Posteriorly, it became useful to assess the mobility of implants and the primary stability of OMI [19, 21]. Percussion must be performed on the OMI head with a small pestle that will rebound at a specific speed depending on stability. During contact, a piezoelectric crystal inside the head of the pestle is deformed, thus creating an electric impulse that reveals the duration of contact, which is converted into stability expressed as PTV, ranging from − 8 to + 50. A lower PTV often indicates better OMI stability. RFA is also a feasible measurement method for OMI stability [22, 23]. A SmartPeg with a permanent magnet is tightened into the implant or OMI. A handpiece emits electromagnetic impulses and a frequency in Hertz is recorded. Following computer-aided data analysis, resonance frequency in Hertz is converted into an “implant stability quotient” (ISQ) value, ranging from 0 to 100. Higher ISQ values indicate better OMI stability [24, 25].

The clinical trials of this systematic review and meta-analysis demonstrated that the LLLT significantly benefited the OMI stability. Promising results regarding this effect has already been shown in animal models [7, 26–28]. Experimental studies facilitate the understanding of biostimulatory mechanisms of photobiomodulation on bone regeneration and inflammation [26]. Garcez et al. demonstrated that a group of animals irradiated with LLLT showed less inflammatory infiltrate and better bone neoformation, with greater organization of collagen fibers, neovascularization, and epithelialization around the OMI. Omasa et al. observed that LLLT accelerated the peri-implant bone formation in rats and that a possible mechanism may be the stimulation of growth and transcription factors involved in the differentiation of osteoblasts, such as bone morphogenetic proteins-2. Goymen et al. and Pinto et al. evaluated the LLLT in rabbits and observed that this therapy increased the OMI stability via peri-implant bone formation. This systematic review and meta-analysis adds to knowledge because only clinical trials (RCTs and Non-RCT) were retrieved providing information with a higher level of scientific evidence.

As regards the secondary outcome of pain, although recent studies have shown that LLLT is effective in reducing pain intensity and duration after dental implant surgery, our study found no difference in pain after OMI placement with photobiomodulation [5, 14, 29]. However, bias might have taken place, as the fixed orthodontic appliance itself may cause the perception of pain reported by patients. In addition, AlSayed Hasan et al. found that the LLLT at intensities of 4 and 16 J did not generate a significant reduction in the levels of orthodontic pain caused by elastomeric separators [30].

Peri-implant inflammation was also assessed in this systematic review and meta-analysis because it is considered one of the main causes of OMI failure. Failures have occurred more frequently in the first weeks after OMI placement, probably because this trauma triggers a local inflammatory response, with an increase of pro-inflammatory cytokines that contribute to tissue destruction [5, 31]. During data extraction, we observed that two RCTs evaluated peri-implantitis. Osman et al. observed a significant reduction in the gingival index around the OMI in the laser group, corroborating literature findings that demonstrate a reduction in the levels of pro-inflammatory cytokines (IL-6 and IL-8) in the peri-implant crevicular fluid of patients who had undergone LLLT [31]. On the other hand, Ekizer et al. did not observe significant changes in the levels of IL-1β between the laser and control groups. This cytokine is relevant in orthodontic movement, as it can enhance osteoclastic activity [32]. What may possibly contribute to this divergence of results in peri-implant inflammation is the difference of patients’ oral hygiene practices [33].

In this systematic review and meta-analysis, only Abohabib et al. assessed clinical success and failure rate of OMI. This clinical trial observed that, despite the increase in the resonance frequency of OMI with laser therapy, the overall clinical success was 78.5% for the treated group and the control group, not suggesting a beneficial effect of LLLT. In contrast, a study that evaluated the effect of LLLT on the OMI success rate in animals, observed that in the laser group the success rate was 80% and higher than the control group, probably due to the anti-inflammatory and biostimulatory effects of this method [27].

The effects of LLLT are related to the treatment protocol, including wavelength, power, energy density, irradiation time, and frequency of treatment [34]. However, by a critical evaluation of the included articles, we did not observe homogeneous parameters in the LLLT protocols, impairing meaningful comparison of the results and demanding a skeptical look at the potential beneficial effects of this approach. This is justified by the fact that there are no univocal standardized guidelines for the use of photobiomodulation for osteoregenerative purposes [35]. However, the increased stability has been achieved in all clinical trials included in this systematic review and meta-analysis.

The wavelength of the LLLT should be highlighted. Two studies used the red laser (RL) [5, 14], three used the infrared laser (IRL) [10, 15, 16], and one used both [6]. Long wavelengths, represented by IRL, penetrate deeper into the tissues, being more suitable to repair bone tissue. RL has more limited penetration power, being indicated to modulate inflammatory processes and stimulate soft tissue repair [36]. However, there is evidence that both can have beneficial effects on bone repair and on inflammatory process [37].

The present study has some limitations. First, only six studies were included. In addition, the LLLT characteristics, OMI dimensions, type of load, and evaluation methods were quite heterogeneous, making the comparison of study results limited. Although more high-quality RCTs with standardized LLLT protocols are highly encouraged, this study provides helpful information to the literature, as it is the first systematic review and meta-analysis of clinical trials assessing the effects of LLLT on OMI stability.

## Conclusions

In general, LLLT has clinical applicability to increase OMI stability. However, due to the limitations of the current study, additional high-quality clinical trials are needed to elucidate the real effects of this therapy on OMI.

## Supplementary Information

**Additional file 1.** Electronic search strategy.

## Data Availability

The datasets used and/or analyzed during the current study are available from the corresponding author on reasonable request.

## Abbreviations

LLLT: Low-level laser therapy
OMI: Orthodontic mini-implants
RCT: Randomized clinical trials
Non-RCT: Non-randomized clinical trials
PRISMA: Preferred Reporting Items for Systematic Reviews and Meta-Analyses
PICOS: Patients, Intervention, Control, Outcome, Study design
ROBINS-I: Risk of Bias in Non-randomized Studies of Interventions
SD: Standard deviation
SMD: Standardized means difference
PVT: Periotest value
RFA: Resonance frequency analysis
