# Supplementary material for: Effects of low-level laser therapy on the orthodontic mini-implants stability: a systematic review and meta-analysis
Source: Prog Orthod. 2021 Feb 15;22:6. doi: 10.1186/s40510-021-00350-y (PMC7882650; doi:10.1186/s40510-021-00350-y)
Supplement: Supplementary file 1 — Additional file 1. Electronic search strategy. [file 40510_2021_350_MOESM1_ESM.docx]

Supplementary Material 1. Electronic search strategy.

| **Databases** | **Strategy** |
| --- | --- |
| Pubmed | (“orthodontic anchorage procedures”[mh] OR “mini implants” OR “mini screws” OR “micro implants” OR “skeletal anchorage” OR “temporary anchorage device” OR “bone screws”[mh]) AND (“lasers”[mh] OR “laser therapy”[mh] OR “low-level light therapy”[mh]) |
| Science Direct | (“orthodontic anchorage procedures” OR “mini implants” OR “mini screws” OR “micro implants” OR “skeletal anchorage” OR “temporary anchorage device”) AND (“lasers” OR “laser therapy” OR “low-level light therapy”) |
| Embase | (“orthodontic anchorage procedures” OR “mini implants” OR “mini screws” OR “micro implants” OR “skeletal anchorage” OR “temporary anchorage device”) AND (“lasers” OR “laser therapy” OR “low-level light therapy”) |
| Scopus | (“orthodontic anchorage procedures” OR “mini implants” OR “mini screws” OR “micro implants” OR “skeletal anchorage” OR “temporary anchorage device”) AND (“lasers” OR “laser therapy” OR “low-level light therapy”) |
| Web of Science | (“orthodontic anchorage procedures” OR “mini implants” OR “mini screws” OR “micro implants” OR “skeletal anchorage” OR “temporary anchorage device” OR “bone screws”) AND (“lasers” OR “laser therapy” OR “low-level light therapy”) |
| Cochrane Library | (“orthodontic anchorage procedures” OR “mini implants” OR “mini screws” OR “micro implants” OR “skeletal anchorage” OR “temporary anchorage device” OR “bone screws”) AND (“lasers” OR “laser therapy” OR “low-level light therapy”) |
| LILACS | (“mini implants” OR “mini screws” OR “micro implants” OR “skeletal anchorage”) AND (“lasers” OR “laser therapy” OR “low-level light therapy”) |
| Google Scholar | (“orthodontic anchorage procedures” OR “mini implants” OR “mini screws” OR “micro implants” OR “skeletal anchorage” OR “temporary anchorage device” OR “bone screws”) AND (“lasers” OR “laser therapy” OR “low-level light therapy”) |
| Clinicaltrials.gov | (“orthodontic anchorage procedures” OR “mini implants” OR “mini screws” OR “micro implants” OR “skeletal anchorage” OR “temporary anchorage device” OR “Bone screws”) AND (“lasers” OR “laser therapy” OR “low-level light therapy”) |
